# Integration of scRNA and bulk RNA-sequence to construct the 5-gene molecular prognostic model based on the heterogeneity of thyroid carcinoma endothelial cell: Five-gene TC prognostic model

**DOI:** 10.3724/abbs.2023254

**Published:** 2024-01-08

**Authors:** Zhaoxian Ni, Shan Cong, Hongchang Li, Jiazhe Liu, Qing Zhang, Chuanchao Wei, Gaofeng Pan, Hui He, Weiyan Liu, Anwei Mao

**Affiliations:** 1 Department of General Surgery Minhang Hospital Fudan University Shanghai 201199 China; 2 Department of Head and Neck Surgery Fudan University Shanghai Cancer Center Shanghai 200032 China; 3 Department of Oncology Shanghai Medical College Fudan University Shanghai 200032 China; 4 Department of Laparoscopic Surgery the First Affiliated Hospital of Dalian Medical University Dalian 116000 China

**Keywords:** thyroid cancer, single-cell RNA-seq, biomarker, prognosis module

## Abstract

Thyroid cancer (TC) is a kind of cancer with high heterogeneity, which leads to significant difference in prognosis. The prognostic molecular processes are not well understood. Cancer cells and tumor microenvironment (TME) cells jointly determine the heterogeneity. However, quite a little attention was paid to cells in the TME in the past years. In this study, we not only reveal that endothelial cells (ECs) are strongly associated with the progress of papillary thyroid cancer (PTC) using single-cell RNA-seq (scRNA-seq) data downloaded from Gene Expression Omnibus (GEO) and WGCNA, but also screen 5 crucial genes of ECs: *CLDN5*, *ABCG2*, *NOTCH4*, *PLAT*, and *TMEM47*. Furthermore, the 5-gene molecular prognostic model is constructed, which can predict how well a patient will do on PD-L1 blockade immunotherapy for TC and evaluate prognosis. Quantitative real-time polymerase chain reaction (qRT-PCR) analysis demonstrates that PLAT is decreased in TC and the increase of PLAT can restrain the migratory capacity of TC cells. Meanwhile, in TC cells, PLAT suppresses VEGFa/VEGFR2-mediated human umbilical vascular endothelial cell (HUVEC) proliferation and tube formation. Totally, we construct the 5-gene molecular prognostic model from the perspective of EC and provide a new idea for immunotherapy of TC.

## Introduction

Among endocrine tumors, TC has the highest incidence rate and is growing at the fastest rate. Thyroid cancer has shown a 300% increase in occurrence over the past three decades [1]. The majority of TC are made up of papillary thyroid cancer (PTC) which can be successfully treated with surgery, radioactive iodine (RAI) ablation, and thyroid stimulating hormone (TSH) suppression, with a 90% ten-year survival rate [2]. About 20% of PTC would have local recurrences and 10% would have distant metastases, mostly in the lung (50%) and bone (25%) [3]. What’s worse, 60%‒70% of these PTC patients with bad prognosis can only live 3–5 years because they are not sensitive to radioiodine therapy [4]. Thus, it is essential to analyze the development of PTC accurately and develop novel therapeutic targets.

The differences in the prognosis of PTC are mainly caused by the heterogeneity of TC. While sharing a shared origin, tumors may exhibit distinct features due to spatial heterogeneity. These variations may have origins in genetic, phenotypic, or behavioral factors [ 5, 6]. The heterogeneity of tumor is associated with inter-tumor heterogeneity and intra-tumor heterogeneity [7], which means that cancer cells and TME cells determine disease progression, therapy efficacy, and the likelihood of escape together. For tumor patients, beyond the standard clinical typing of cancer cell somatic mutations, only comprehensive analysis of the entire tumor ecosystem can provide individualized treatment [ 8, 9]. However, in the past years, many studies focused on the tumor cells and ignored the role of TME in the development of TC. Meanwhile, prognostic models of thyroid cancer have been established based on ferroptosis-related gene, up-regulated glycolysis-related genes, pyroptosis-related genes, lipid metabolism-related genes and so on [ 10‒ 13]. Considering the current situation, it is of great significance to analyze the progress of PTC and establish the prognostic model from the perspective of TME. In the meantime, as scRNA-seq developed, we can analyze the cell heterogeneity more intuitively and reveal the internal mechanism of the development of tumor.

In the present study, we constructed 14 cell clusters and 9 co-expression modules using scRNA-seq data of 7 primary tumor samples in GSE184362 and bulk-RNA-seq data from TCGA database. Through the correlation analysis, we identified C10 cluster belonging to EC had a strong relationship with the progress of tumor, and selected 5 genes of C10 as crucial genes through Lasso Cox regression and step AIC. The 5-gene molecular prognostic model was constructed, which can effectively assess the patients’ prognoses.

## Materials and Methods

### Collection of data from GEO database and TCGA database

The GSE184362 dataset, downloaded from the GEO ( https://www.ncbi.nlm.nih.gov/) database, comprises scRNA-seq data from 7 primary tumors, 6 para-tumors, 8 metastatic lymph nodes and 2 subcutaneous metastatic loci. This study only used data from 7 primary tumors. Then we downloaded GSE6004 (14 tumor tissues and 4 normal tissues), GSE33630 (45 normal tissues and 60 tumor tissues) and GSE60542 (58 tumor tissues and 34 normal tissues) from the GEO database, which were processed as follows: the gene ID was converted into the gene symbol. Genes were deleted if several genes were eliminated by a probe. The expression of gene was replaced by the average value of a gene when several probes detect the gene. Publicly available clinical data and information on gene expression were retrieved from TCGA database ( https://portal.gdc.cancer.gov/).

### Quality control of data

The Seurat R package was used to import and process the raw gene expression matrices of 7 primary tumors obtained from GSE184362 [14]. The process was as follows: first, cell filtration was carried out based on one of these three criteria: (1) At least three cells express this gene, and each cell expresses at least 250 genes. (2) The Percentage Feature Set function is used to calculate the percentages of mitochondria and rRNA and ensure that each cell expresses more than 500 genes but less than 5500 genes. Mitochondrial genes constitute less than 35% of the genome. (3) At least 1000 unique molecular identifiers (UMIs) are present in each single cell. Then, the three samples were combined and merged for further analysis.

### Construction of cell clusters

The gene expression profiles of the preprocessed cells were then normalized using “Log Normalize” R package. The Find Variable Features function [Variable characteristics are identified based on variable-stabilized transformation (“VST”)] was used to find highly variable genes. Then, the function “RunPCA” was used to carry out the principal component analysis (PCA). FindNeighbors and FindClusters functions were used to cluster cells (resolution=0.1, dim=50), and t-distributed stochastic neighbor embedding (t-SNE) was used to visualize them. FindAllMarkers function was used to identify the marker genes of each cluster (logFC=0.35, min pct=0.15, p-adj<0.05) to determine which cell type it belongs to.

### Co-expression network construction by WGCNA

The samples were hierarchically clustered. First, the samples were clustered using Pearson’s correlation coefficient and a gene co-expression network was constructed using the R package termed “WGCNA” to screen the crucial gene co-expression modules [15]. The gene co-expression network was proved that it fit the scale-free network. The logarithm of the node with connection degree K (log(k)) had a negative correlation with the logarithm of the probability of the node (log(P(k))), which is higher than 0.85. Furthermore, the adjacency matrix was converted into a topological overlap matrix (TOM). Next, genes were clustered into gene modules using average-linkage hierarchical clustering and dynamic tree clipping based on TOM. Each module contains at least 30 genes (min Module Size=30). Finally, the eigengenes of each module were counted by turns and the modules were subject to cluster analysis, which can merge the modules which are close to each other into a new module (height=0.25, deep split=2, min module size=30).

### Crucial gene module GO and KEGG enrichment analysis

The online Gene Ontology (GO) platform WebGestalt R was used to conduct a GO term enrichment analysis, which takes biological processes (BP), cellular components (CC) and molecular functions (MF) into account. To determine which pathways are most likely to enrich in a crucial gene module, we performed an online platform David-based Kyoto Encyclopedia of Genes and Genomes (KEGG) pathway enrichment analysis. FDR<0.05 was set as statistically significant.

### Pseudotime analysis

Pseudotime analysis was used to predict cell changes over time by constructing intercellular change trajectories. CytoTRACE is a computational framework to predict differentiation states from scRNA-seq data using the measure of transcriptional diversity [16].

### Construction and evaluation of the prognosis-related signature

To further select and delete relevant genes, R package “glmnet” was used to conduct LASSO Cox regression [17]. As the lambda climbed, so did the number of independent variable coefficients that tended to zero. We built the prognosis-related signature using 10-fold cross-validation and examined the confidence intervals for each lambda. The MASS package’s step AIC function was used to delete variable from the most complex module and reduce AIC. The model performed better with a smaller number, indicating that it needs fewer parameters to achieve an adequate fitting degree.

### Construction of the nomogram model

Using the prognosis-related signature and clinicopathological parameters, we built the nomogram model to better predict the prognosis of patients with thyroid cancer. Calibrate Plot was drawn to evaluate the precision of the nomogram model’s calibration. Calibration was the difference between predicted outcome events and actual outcome events. Decision curve analysis (DCA) was used to analyze the clinical benefit, whether it benefits in clinical application.

### Single-sample gene set enrichment analysis (ssGSEA)

To quantify the absolute enrichment of a gene set across all samples, the ssGSEA algorithm uses a ranking-based scoring system. This study used GSVA package in R to perform ssGSEA [18]. Pearson’s method was used to calculate the correlation of the ssGSEA scores.

### Cell culture and transfection

The method and material of cell culture and transfection were described previously [19]. In brief, pEGFP-C1-PLAT was purchased from Genepharma (Shanghai, China). The expression vector pEGFP-C1-PLAT or pEGFP-C1 was transfected into IHH4 cells by using Lipofectamine 3000 reagent (Invitrogen, Carlsbad, USA). Next, we performed RNA isolation and qRT-PCR analysis using the method as described previously [19].

### Wound healing assay

Six-well plates were planted with IHH4 cells. When the cells formed monolayers of compact cells, a 200-μL plastic pipette tip was used to make scratch wounds on the monolayers and cell debris were washed away with PBS. Then the plates were cultured with serum-free 1640 medium. Images of wounds were taken at the specified time points using an inverted microscope (Olympus. Tokyo, Japan) and analyzed with Image J (NIH, Bethesda, USA). All assays were performed in triplicate.

At 100% confluence, HUVECs (ATCC, Manassas, USA) were scratched vertically with a pipet tip for the wound healing assay. Next, the medium was replaced by tumor-conditioned medium (TCM). After two days, photographs were taken once every 24 h to track the HUVECs’ migration distance from the wounds. ImageJ software was used to calculate the migration rate of HUVECs.

### Tube formation assay

Thyroid cancer cell culture medium was collected, centrifuged, and filtered after cells were cultured for 48 h in serum-free DMEM medium. . After the wells of the precooled 96-well plate were coated with 50 μL of growth-factor-reduced Matrigel (ABW 082704; ABW, Shanghai, China), the plate was incubated at 37°C for 45 min. When HUVEC cells were 70% to 80% confluence, they were trypsinized and resuspended in DMEM containing 10% FBS and counted. Cells (50 μL) were plated into each well of the plate at 30,000 cells/well. After incubation in an incubator at 37°C for 4 h, blood vessel formation was observed. Then, fluorescence staining was performed to determine tube formation. Calcein diluted in serum-free medium (50 μL) was added in each well at a final concentration of 6.25 μg/mL and then incubate at room temperature for 30 min in the dark. Finally, immunofluorescence imaging were performed after 3 times wash with PBS. The “Angiogenesis Analyzer” plugin for Image J was used to count the number of tubes [20], and the experiment was performed in triplicate.

### Statistical analysis

R version 3.5.3 and GraphPad Prism version 8.01 (GraphPad Software, La Jolla, USA) were used for all statistical analyses. The significance level for the Student’s *t*-test used to evaluate the correlation between the experimental and control groups was set at *P*<0.05.

## Results

### Construction of cell clusters and cell annotation

**Figure 1:**
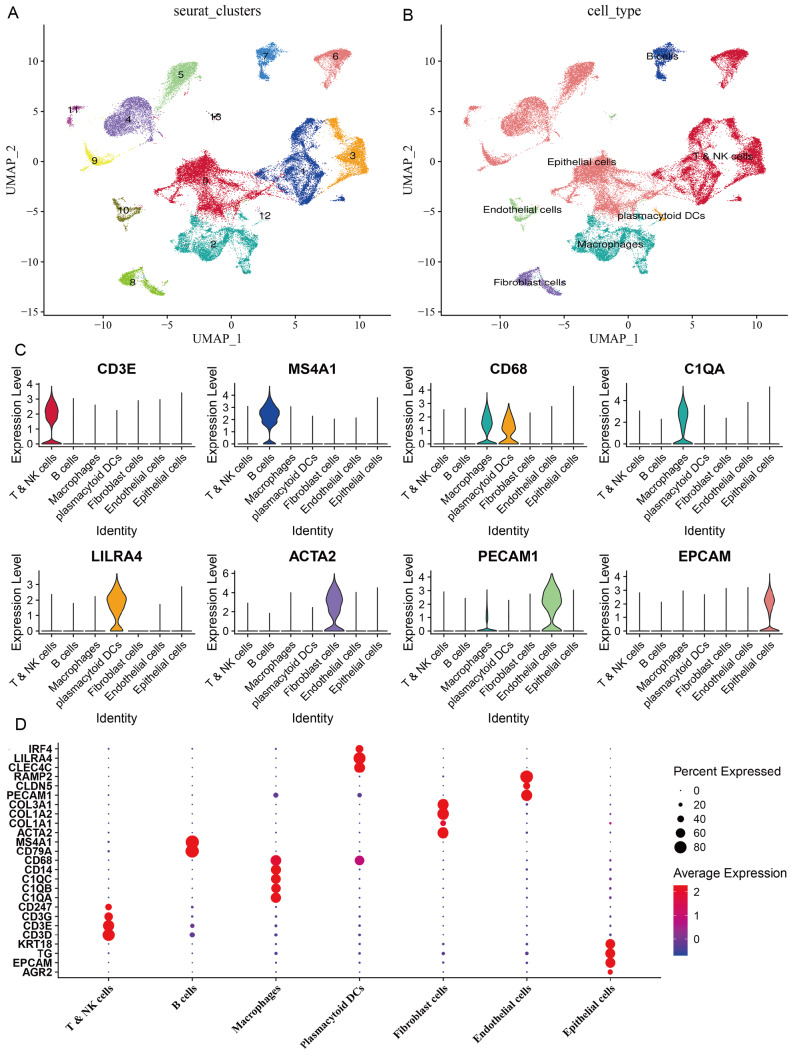
Figure 1 Construction of cell clusters and screening of marker genes (A) The UMAP plot of cells showing 14 cell clusters. (B) The UMAP plot of cells showing annotated cell clusters. (C) The expressions of the first marker genes in 7 cell types. (D) The expressions of marker genes in 7 cell types. The size of the dot represents the fraction of cells in a given cluster in which the gene is detected. The color of the dot represents the average expression score of the cells within a given cluster.

To comprehensively understand the differences of tumor cells, cells in the TME in PTC of different state, we chosen the scRNA-seq data from 7 primary tumors which can represent the tumor evolution: intrathyroidal tumor (PTC9), extrathyroidal extension (PTC1), lateral neck metastasis (PTC2 and PTC3), extrathyroidal extension and lateral neck metastasis (PTC8), and distant metastasis (PTC5 and PTC10). After the stringent quality filtering ( Supplementary Figure S1) and using the Find Neighbors function and Find Clusters function, the cells were divided into 14 cell clusters ( Figure 1A). Next, based on the human cell marker genes obtained from the website for human cell marker ( http://biocc.hrbmu.edu.cn/CellMarker) and Single Cell Base ( http://cloud.capitalbiotech.com/SingleCellBase/), we annotated 14 cell clusters into 7 cell types: epithelial cells, T/NK cells, macrophages, B cells, fibroblast cells, endothelial cells, and plasmacytoid DCs ( Figure 1B‒D and Table 1). Among them, C0, C4, C5, C9 and C11 belong to epithelial cells marked by EPCAM and TG [21]; C1, C3 and C6 belong to T/NK cells with the markers of CD3D [22]; and C10 and C13 belong to endothelial cells with markers of CLDN5, PECAM1 and COL3A1 [ 21, 23].

**Table:** **Table 1** Subgroup definition information

Seraut_cluster	Cell_type
C0	Epithelial cells
C1	T/NK cells
C2	Macrophages
C3	T/NK cells
C4	Epithelial cells
C5	Epithelial cells,
C6	T/NK cells
C7	B cells
C8	Fibroblast cells
C9	Epithelial cells
C10	Endothelial cells
C11	Epithelial cells
C12	Plasmacytoid DCs
C13	Endothelial cells

### Screening of different cell clusters

Then we screened the crucial cell clusters which are differently distributed in normal tissues and tumor tissues using bulk RNA-seq data from TCGA dataset through CIBERSORT R package [24]. As we can see, all cell clusters have significant difference between tumor tissue and normal tissue except C3 and C8 ( Supplementary Figure S2), which means that all 8 cell types are associated with the occurrence and progress of PTC.

### Identification of C10 as a crucial cell cluster associated with the progress of PTC

First, weighted gene co-expression network analysis (WGCNA) was used to mine co-expression coding genes and co-expression modules. We constructed 6 gene modules with the soft threshold of 8 using the gene expression patterns in 487 TCGA cohort tumor samples ( Supplementary Figure S3A–C), among which the red module is significantly correlated with the tumor. Next, we performed GO analysis of the biological significance and pathway of red modules. There are 241 GO terms with significant difference in BP (FDR<0.05) in the pathway of blood vessel morphogenesis and angiogenesis ( Supplementary Figure S4A). For GO CC analysis, the top two significantly enriched terms of 28 GO terms (FDR<0.05) are extracellular matrix structural constituent and growth factor binding ( Supplementary Figure S4B). There are 9 GO terms with significant difference in MF (FDR<0.05) in the pathway of extracellular matrix and collagen-containing extracellular matrix ( Supplementary Figure S4C). Then through KEGG Enrichment Analysis ( Supplementary Figure S4D), the genes are enriched in 27 pathways, which are mainly associated with PI3K-Akt signaling pathway, Rap l signaling pathway and so on. Thus, the red module is mainly associated with tumorigenesis. The relationship between gene modules and the abundance of these clusters was analyzed further. C10 was identified as the crucial cell cluster which is closely related to the red module ( Supplementary Figure S3D). Given that C10 belongs to endothelial cells together with C13, we performed Pseudotime Analysis of C10 and C13 using CytoTRACE package in R. The CytoTRACE of C13 is lower than that of C10 ( Supplementary Figure S5A), which means that C10 is of lower differentiation than C13. Furthermore, we got related genes to distinguish the differentiation of Endothelial cell ( Supplementary Figure S5B) and UMAP were used to show that C10 is of low differentiation and C13 is of different differentiation ( Supplementary Figure S5C,D).

### Screening of crucial genes and construction of the prognosis-related signature

Considering that there is a strong relationship between C10 and red module, we overlapped the genes of red module and marker genes of C10 ( Supplementary Figure S6A). There are 128 genes in common, which are significantly associated with thyroid cancer. To further analyze these 128 genes, the TCGA cohort’s 487 tumor samples were split into a “training” and “validation” dataset using “do by function” in R. Sampling fraction was 0.8. The training dataset included 390 samples and the validation dataset included 97 samples ( Table 2). The chi-square test was used to analyze the differences between the two datasets. Statistical analysis revealed no significant difference ( *P*>0.05). Subsequently, the “coxph function” of survival package was used to perform univariate Cox proportional-hazards regression analysis ( *P*<0.05) to find crucial genes associated with survival in these 128 genes ( Supplementary Table S1). To further select and delete relevant genes, LASSO Cox regression was used to analyze the trajectory of change for each variable ( Supplementary Figure S6B). Next, we built the prognosis-related signature and analyzed the confidence intervals for each lambda using 10×fold cross-validation. The optimal model parameters are lambda=0.0118 ( Supplementary Figure S6C). Finally, 5 genes ( *CLDN5*, *ABCG2*, *NOTCH4*, *PLAT*, and *TMEM47*) were chosen to construct the prognosis-related signature by step AIC. The signature is as follows: risk score=‒0.747×CLDN5‒0.807×ABCG2+1.477×NOTCH4‒0.281×PLAT‒0.476×TMEM47.

**Table:** **Table 2** Information of TCGA training and test samples

Clinical features	TCGA-training	TCGA-test	*P* value
OS			1
0	354	88	
1	36	9	
T stage			0.388
T1	114	27	
T2	130	33	
T3	132	28	
T4	14	7	
Stage			0.827
I	223	57	
II	42	7	
III	85	19	
IV	38	12	
Gender			0.6803
Male	288	69	
Female	102	28	
Age			0.5707
≤50	228	53	
>50	162	44	

### Assessment and validation of the 5-gene molecular prognostic model

**Figure 2:**
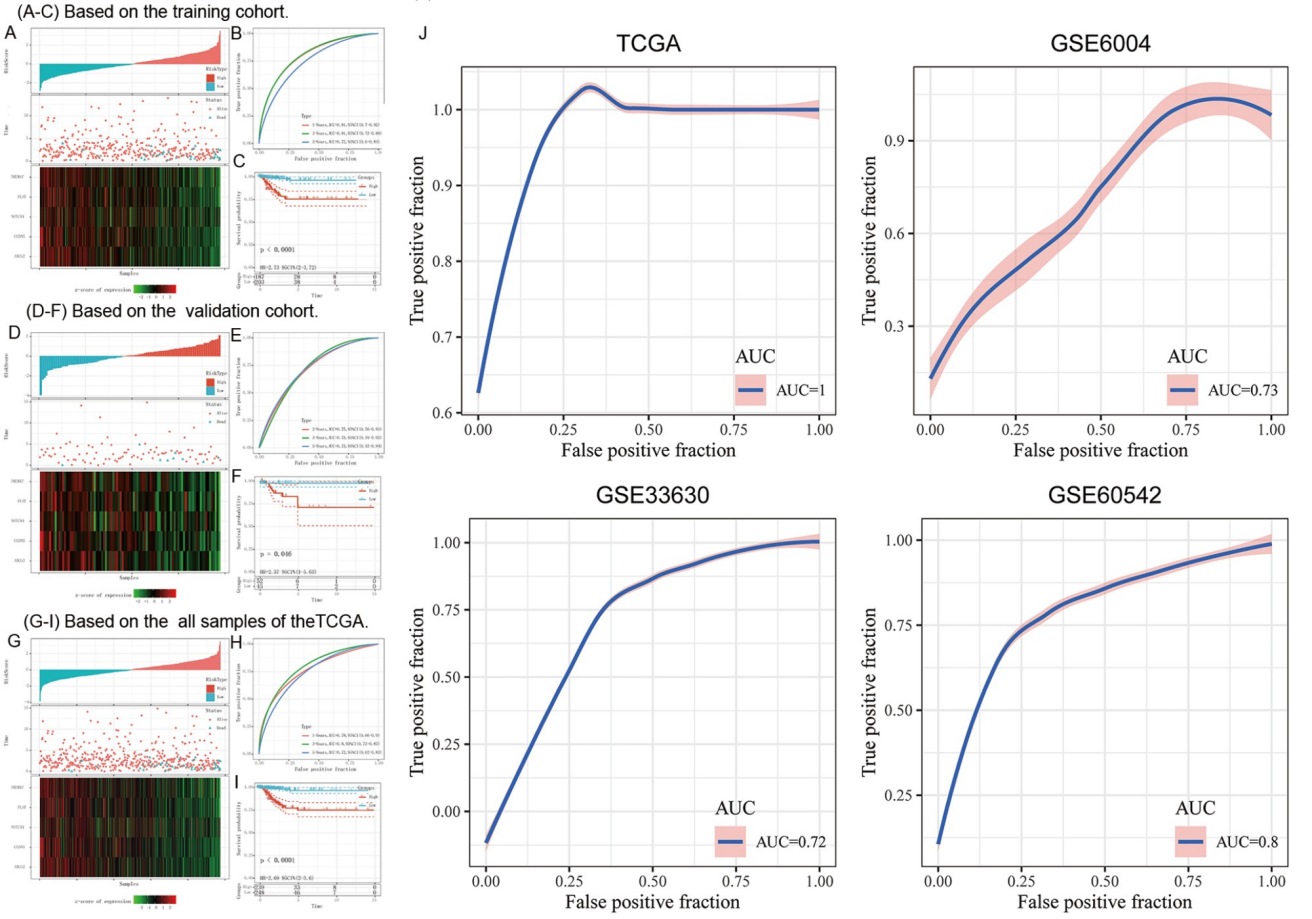
Figure 2 Assessment and validation of the 5-gene molecular prognostic model (A) Based on the 5-gene molecular prognostic model, the tumor samples of training cohort in TCGA classifier were classified into low-risk group and high-risk group. The overall survivals of tumor samples, and their survival status. A heatmap of five crucial genes in the training cohort. (B) The distribution of time-dependent ROC curves. (C) K-M curve of training cohort in TCGA classifier. (D) Based on the 5-gene molecular prognostic model, the tumor samples of independent validation cohort in TCGA classifier were classified into low-risk group and high-risk group. The overall survivals of these samples, and their survival status. A heatmap of five crucial genes in the validation cohort. (E) The distribution of time-dependent ROC curves. (F) K-M curve of validation cohort in TCGA classifier. (G) Based on the 5-gene molecular prognostic model, all tumor samples of TCGA classifier were classified into low-risk group and high-risk group. The overall survivals of all samples, and their survival status. A heatmap of five crucial genes. (H) The distribution of time-dependent ROC curves. (I) K-M curve of all samples in TCGA classifier. (J) Validation of SVM based on TCGA classifier. ROC curves of TCGA, GSE6004, GSE33630, and GSE60542 in GEO. ROC, receiver operator characteristic. AUC, the area under the curve.

Through measuring the expressions of the 5 genes in training dataset from TCGA cohort, we tallied up each sample’s risk rating and plotted the results ( Figure 2A). Subsequently, the “time ROC” R package was used to execute a time-dependent receiver-operating characteristic (ROC) curve analysis on the risk score. We respectively analyzed the classification efficiency of prognosis prediction at one, three and five years. ROC curve analysis ( Figure 2B) demonstrated that the 5-gene molecular prognostic model may perform well at predicting 1-year OS, 3-year OS, and 5-year OS. The area under the ROC curve (AUC) values are 0.81, 0.81, and 0.72 respectively. Finally, risk score was performed, which was finally divided into 2 groups, high-risk group (risk score>0) and low risk group (risk score<0). Kaplan-Meier curves (K-M curves) ( Figure 2C) showed the sample in the high-risk group had a shorter OS compared to those in the low-risk group (HR=2.73, 95% CI=2.00–3.72, *P*<0.0001).

### Validation of the 5-gene molecular prognostic model in TCGA dataset and GEO cohort

To validate the 5 gene molecular prognostic model, we used these gene expression data from validation dataset in TCGA cohort and the whole TCGA dataset. We also tallied up each sample’s risk score and drew the risk score distribution ( Figure 2D) and performed ROC curves and K-M curves. ROC curve analysis ( Figure 2E) showed that the 5 gene molecular prognostic model performed well at predicting 1-year OS, 3-year OS, 5-year OS. The AUC values are 0.75, 0.75, and 0.73 respectively, which means that the module has good predictive value. K-M curves ( Figure 2F) also showed that the high-risk sample has a lower OS than the low-risk sample (HR=2.73, 95% CI=2.00–3.72, *P*<0.0001). Furthermore, we did the same validation on the entire dataset ( Figure 2G). The AUC values of 1-year OS, 3-year OS, 5-year OS are 0.78, 0.8, and 0.72 respectively, which also identifies the sensitivity of the module. K-M curves ( Figure 2H) showed that the low-risk sample had a longer OS than the high-risk sample ( Figure 2I).

To further validate the 5 gene molecular prognostic model, we put all TCGA dataset as training dataset. We constructed support vector machine (SVM) classification model using Tenfold cross-validation based on the 5 gene molecular prognostic model. The accuracy of classification reached 100%. Meanwhile, GSE6004, GSE33630 and GSE60542 downloaded from GEO were used as validation dataset. We constructed the SVM of three validation datasets based on the 5-gene molecular prognostic mode and found that the AUC values are greater than 0.7 ( Figure 2J).

### Risk score on different clinical characteristics

**Figure 3:**
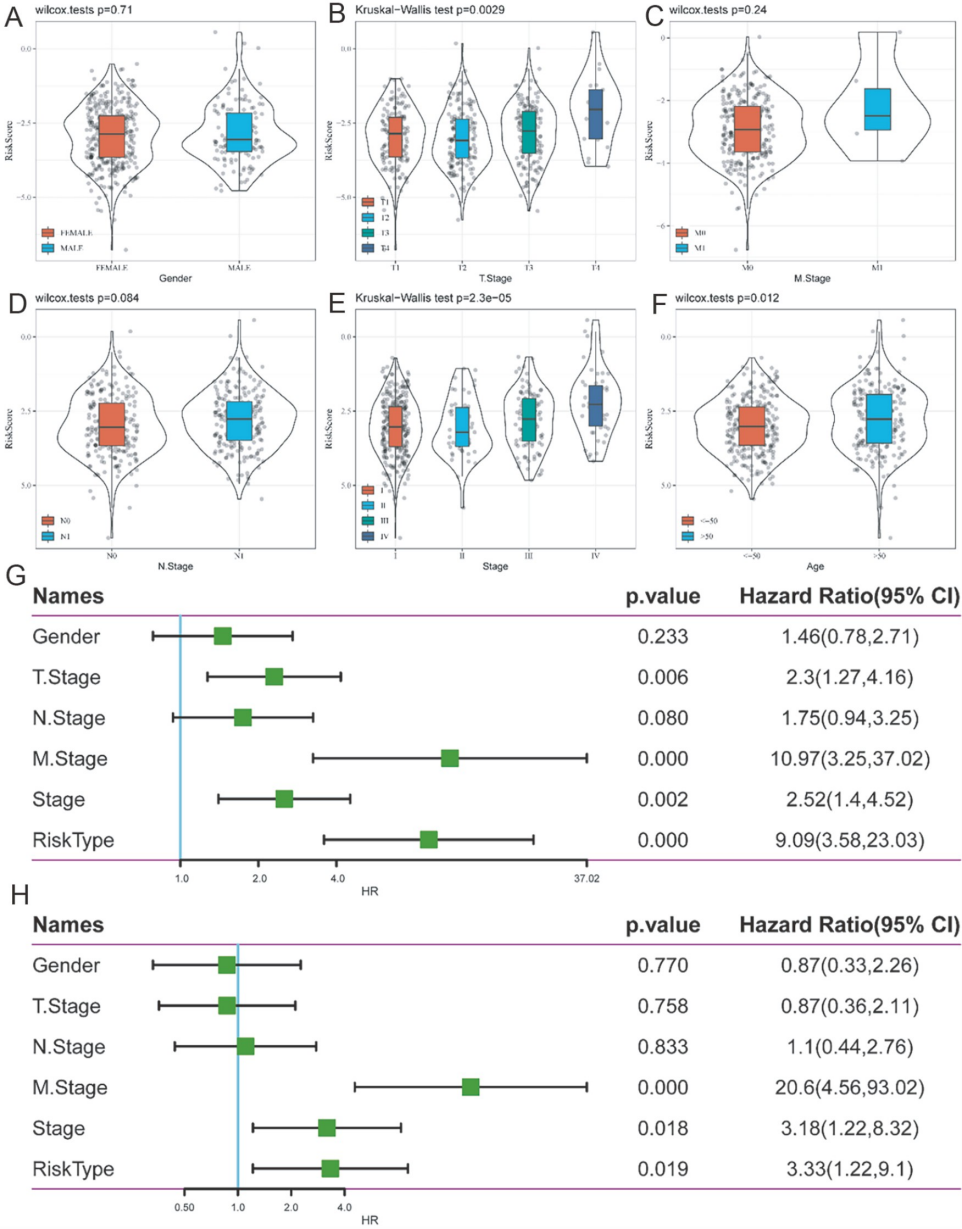
Figure 3 The relationship between the 5-gene molecular prognostic model and clinical features (A) Risk score on female and male. (B) Risk score on T stage (P<0.05). (C) Risk score on M stage. (D) Risk score on N stage. (E) Risk score on stage (P<0.05). (F) Risk score on age (P<0.05). (G) Forest plot of univariate Cox regression analysis in TCGA cohort. (H) Forest plot of multivariable Cox regression analysis in TCGA cohort.

We used Student’s *t*-test to evaluate the distributions of risk scores across sex, T stage, N stage, M stage, and age to analyze the association between risk score and clinical features ( Figure 3A‒F). We found that the risk score is differently distributed in T stage, stage and age ( *P*<0.05). Next, the entire TCGA dataset was subjected to a univariate and multivariate Cox regression analysis, taking variables such as gender, stage, T stage, N stage, M stage, and risk type into account ( Figure 3G,H).

### Construction and assessment of the nomogram model

**Figure 4:**
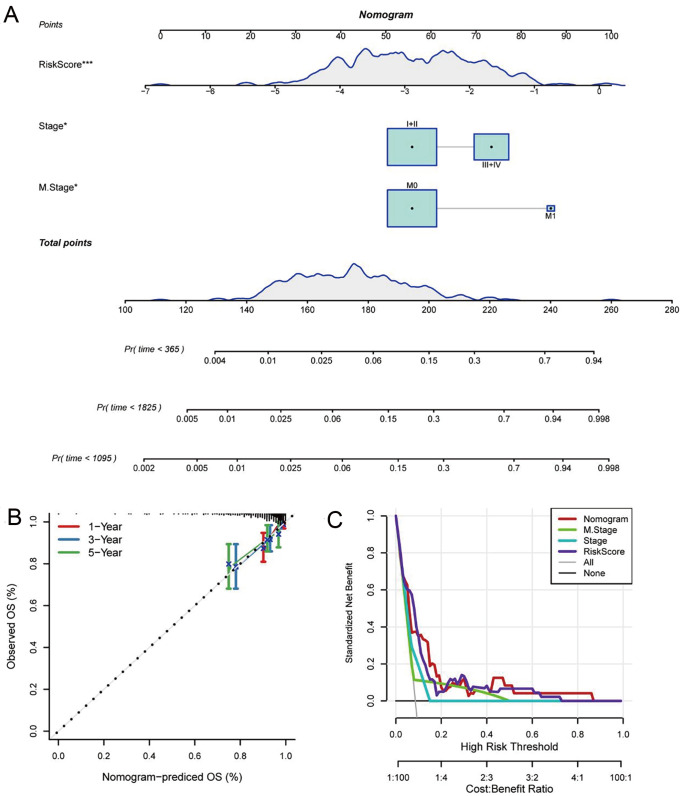
Figure 4 The nomogram tool for predicting survival rate in TCGA classifier (A) The nomogram tools. (B) Calibration curve of nomogram. (C) DCA of stage, S stage, RS, and nomogram.

To help doctors statistically anticipate their patients’ chances of surviving 1, 3, and 5 years after their PTC diagnosis, we created a prognostic nomogram based on multivariate Cox regression of 5-gene signature and clinical features (M stage and stage) in the TCGA cohort for training ( Figure 4A). The Calibrate plot of the nomogram model showed a good concordance ( Figure 4B). We observed that the risk score is the most important factor in determining survival, suggesting that the 5-gene molecular prognostic model provides more accurate prognostic information. In addition, we plotted DCA diagrams of stage, M stage, risk score and nomogram ( Figure 4C). It showed that the nomogram model could benefit in clinical application with threshold of 0.05‒0.84. To sum up, the nomogram model has a good predictive effect.

### Pathways potentially regulated by risk score

To learn more on pathways that the 5-gene prognosis module regulates TC, ssGSEA analysis was performed to calculate the score of each sample in different functions by R package GSVA. Samples were downloaded from TCGA dataset.

We then determined the link between these characteristics and the risk score based on ssGSEA of each sample in all functions. The functions with correlation ≥0.3 were selected ( Supplementary Figure S7A), from which we found that 6 pathways had negative correlation with risk score and 6 pathways had positive correlation with risk score. Then we performed KEGG enrichment analysis. According to their enrichment scores ( Supplementary Figure S7B), 6 pathways positively associated with risk score were sorted by influence as follows: base excision repair, one carbon pool by folate, homologous, DNA replication, nucleotide excision repair and mismatch repair; 6 pathways negatively associated with risk score were sorted by influence as follows: melanogenesis, hedgehog signaling pathway, calcium signaling pathway, glycerolipid metabolism, proximal tubule, and type II diabetes mellitus.

### Analysis of 5 EC-marker genes

**Figure 5:**
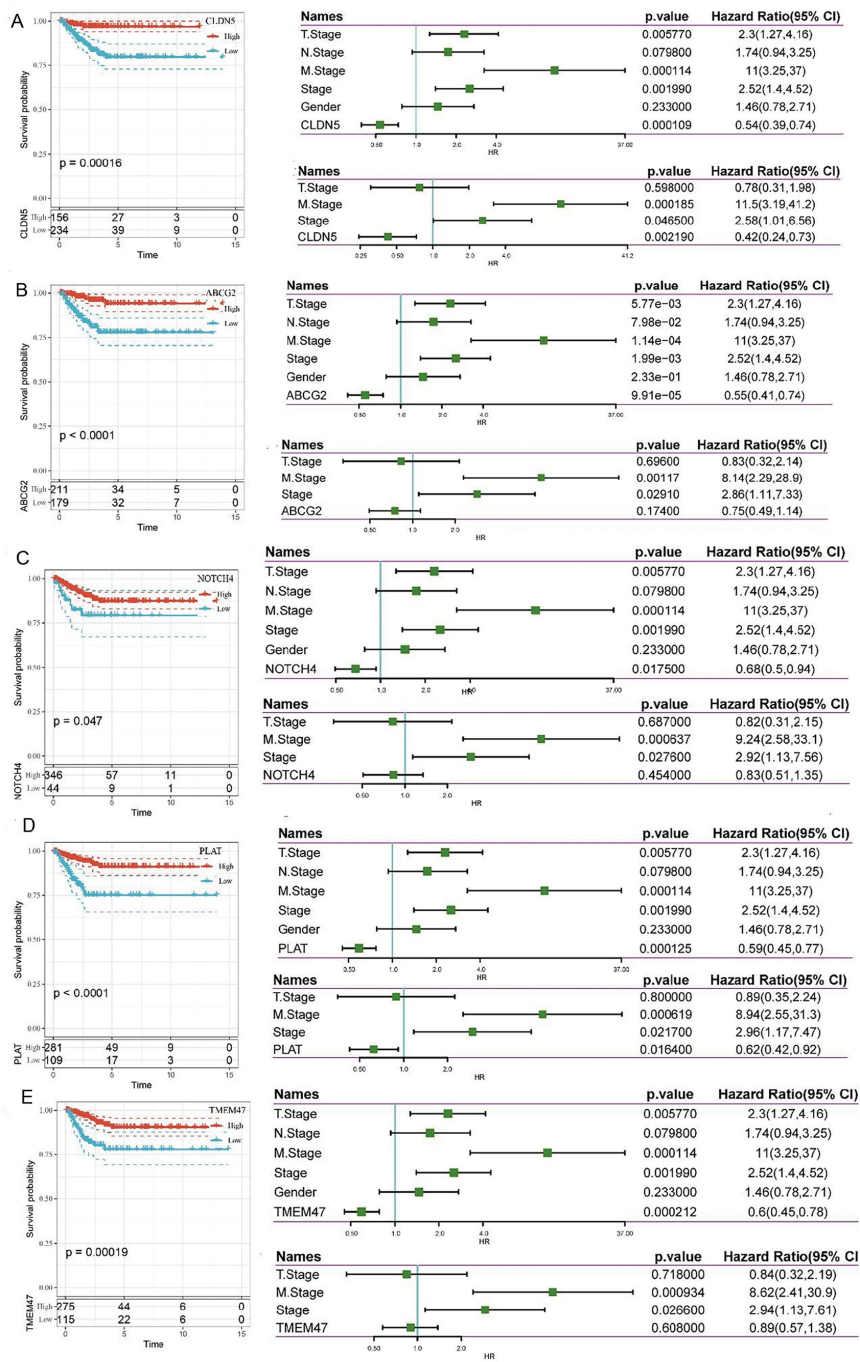
Figure 5 The relationship between 5 single gene and clinical characteristics

We performed univariable and multivariable Cox regression of 5 EC-marker genes to analyze the connection between each gene and the prognosis of TC. Univariable Cox regression showed that all genes are strongly associated with the prognosis: *ABCG2* (HR=0.55, 95% CI=0.41‒0.74, *P*<0.05), *NOTCH4* (HR=0.68, 95% CI=0.5‒0.94, *P*<0.05), *TMEM47* (HR=0.6, 95% CI=0.45‒0.78, *P*<0.05), *CLDN5* (HR=0.54, 95% CI=0.39‒0.74, *P*<0.05), and *PLAT* (HR=0.59, 95% CI=0.45‒0.77, *P*<0.05). However, multivariable cox regression showed only CLDN5 (HR=0.42, 95% CI=0.24‒0.73, *P*<0.05) and PLAT (HR=0.62, 95% CI=0.42‒0.92, *P*<0.05) can be used as independent prognostic factors. K-M curves revealed that samples in the group with high gene expression had prolonged OS than those in the group with low gene expression ( Figure 5). As we can see, the gene module shows that risk score is increased with the increase of NOTCH4, which is contrary to the result. We consider that NOTCH4 may be affected by other genes and reversed.

### The relationship between the 5-gene prognosis module and immunotherapy

ECs can interact with immune cells in the TME and may become a new target for immunotherapy [25]. In our study, we further explored the interactions between them using the CIBERSORT R package. The 22 immune cells were differently distributed not only in each sample but also between those at higher risk and lower risk ( Supplementary Figure S8A,B). Regulatory T cells (Tregs) were higher but Macrophages M2 were lower in the high-risk group. Immune checkpoints are control points that can either increase or decrease an immunological response, which can impact T-cell activation and peripheral tolerance maintenance [26]. We found that 21 (44.68%) checkpoint genes of 47 immune checkpoints differed substantially between groups at high and low risk ( Supplementary Figure S8C). Using TIDE ( http://tide.dfci.harvard.edu/), we evaluated the prospective clinical effects of immunotherapy in our defined molecular subtypes. When the TIDE prediction score rises, so does the likelihood of immune escape, and the possibility of patients who can benefit from immunotherapy is decreased. In the TCGA dataset, we found that those at low risk have a low TIDE score, whereas those at high risk have a high TIDE score ( Supplementary Figure S9A), indicating that immunotherapy is most effective in group at low risk. Meanwhile, we compared the differences in predicted T-cell dysfunction scores, T-cell rejection scores, and myeloid derived suppressor cells (MDSCs) between groups. The dysfunction and exclusion score was higher in the high-risk group than in the low-risk group, but the MDSCs score was lower ( Supplementary Figure S9B-D). Subsequently, we investigated the risk score’s ability to predict patient response to ICB treatment. In the anti-PD-L1 cohort (IMvigor210 cohort), we discovered that the risk score was drastically lower in the CR/PR group than in the SD/PD group ( Supplementary Figure S9E). In addition, the CR/PR ratio was lower in the group with high risk than in the group with low risk ( Supplementary Figure S9F), meaning that the PD-L1 immunotherapy is more effective in the low-risk group than in the high-EC risk group., which may be because more MDSCs in the lower-risk group inhibit immune response and PD-L1 immunotherapy increase the immune response. Based on the above analysis results, we believe that PD-L1 immunotherapy may not have a satisfactory result in the group at high risk.

### Overexpression of PLAT inhibits the migratory capacity of thyroid cancer cells

**Figure 6:**
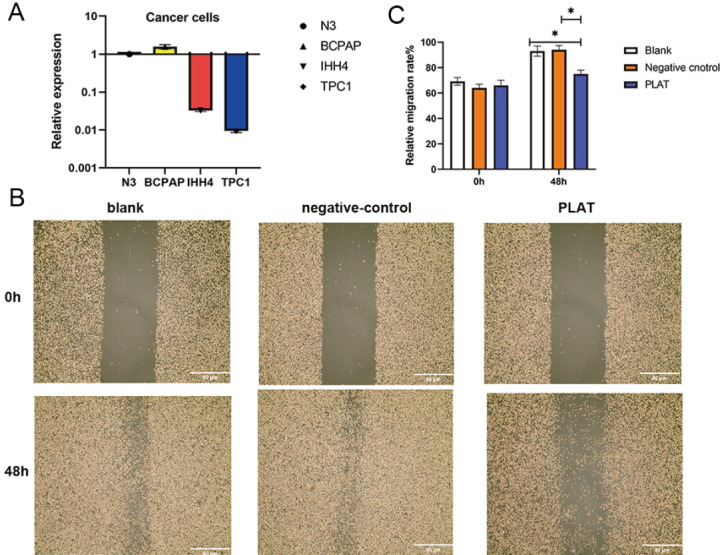
Figure 6 PLAT suppresses the migration ability of thyroid cancer cells (A) The different expressions of PLAT in N3, BCPAP, TPC1 and IHH4 cell lines. (B) The results of wound healing assays. (C) Bar graph showing the size of the scratch area before and after 48 h in 3 cell groups.

Considering that only CLDN5 and PLAT can be used as independent factors of prognosis and there were few studies on *PLAT* in PTC, we focused on this gene. Figure 6A showed that PLAT had differential expression in BCPAP, TPC1 and IHH4 cancer cell lines. In both IHH4 and TPC1 cells, PLAT expression was quite low. To learn more about how PLAT affects thyroid cancer cells, we conducted cell scratch experiments in IHH4 cell lines. Compared to the experimental group, the control group showed a marked decrease in the fusion area of cell scratches ( Figure 6B,C), indicating that PLAT may decrease the migratory ability of TC cells.

### PLAT inhibits thyroid cancer angiogenesis through VEGFa/VEGFR2

**Figure 7:**
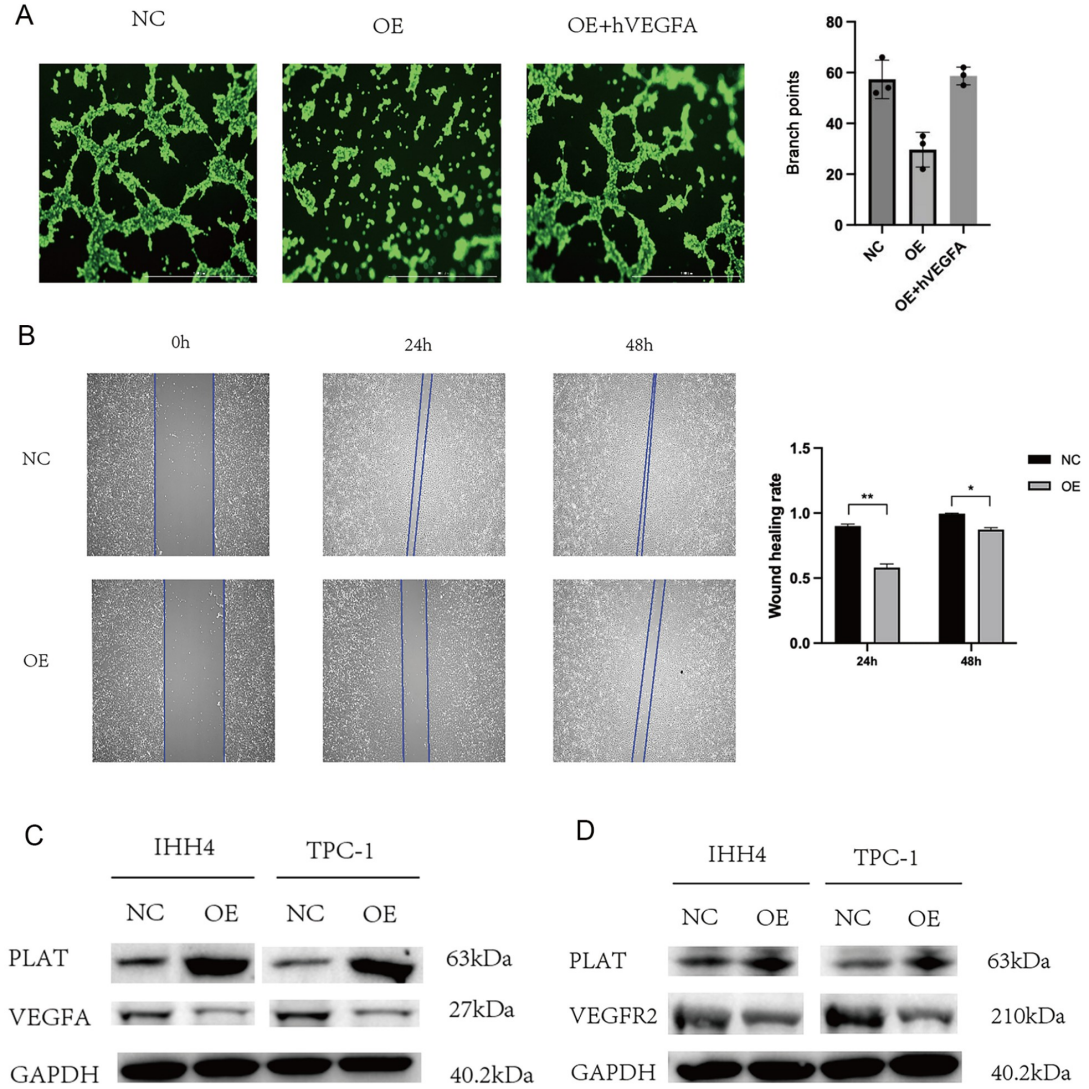
Figure 7 PLAT inhibits thyroid cancer angiogenesis through VEGFa/VEGFR2 (A) Tube formation ability of HUVECs cultured for 4 h in the TCM from NC, OE and OE+hVEGFA. (B) Wound healing ability of HUVECs after 24 h and 48 h in the TCM from NC and OE. (C) Western blot analysis showed that VEGFA protein expression was downregulated in OE cells. (D) Western blot analysis showed that VEGFR2 protein expression was downregulated in HUVECs in TCM from transfected IHH4 and TPC-1 cells overexpressing PLAT. Magnification: 50×. *P<0.05, **P<0.01.

Considering that PLAT was reported to promote tumor angiogenesis in lung cancer [27] but low expression of PLAT indicates poor prognosis in breast cancer [28], wound healing assay and HUVEC tube formation assay were carried out to validate the relationship between angiogenesis and PLAT in thyroid cancer. Overexpression of PLAT in IHH4 cells caused HUVECs to produce fewer and smaller tubes compared to untreated cells ( Figure 7A). In addition, the migration rate of HUVECs in the TCM derived from IHH4-PLAT cells was lower than in the TCM derived from IHH4-Vec cells ( Figure 7B). However, HUVECs migrated faster in the TCM derived from IHH4-Vec cells than they did in the TCM derived from IHH4-PLAT cells ( Figure 7B). These findings demonstrated that PLAT impeded the angiogenesis of thyroid carcinoma *in vitro*. Furthermore, considering that VEGF has a strong relationship with angiogenesis, we analyzed the relationship between PLAT and VEGF to examine the mechanism by which PLAT inhibits the angiogenesis of TC. We detected the expression of VEGFa in the IHH4 and TPC-1 cells and the results revealed that VEGFa expression was significantly decreased in the IHH4 and TPC-1 cells overexpressing PLAT compared to that in normal cells ( Figure 7C). And when the exogenous human VEGFA (hVEGFA) was increased, the inhibition of PLAT was restrained ( Figure 7A), suggesting that PLAT inhibits thyroid cancer angiogenesis through VEGFa. Secreted VEGFa has been reported to induce angiogenesis by forming a complex with VEGFR2 on the membrane of endothelial cells [29]. Therefore, we further evaluated the protein levels of VEGFR2 in HUVECs that had been cultured in TCM from transfected IHH4 and TPC-1 cells. The expression of VEGFR2 was significantly decreased in HUVECs cultured in TCM from transfected IHH4 and TPC-1 cells overexpressing PLAT compared with that obtained in TCM from IHH4 and TPC-1 normal cells ( Figure 7D). Previous studies have revealed that VEGFR2 can induce angiogenesis [ 30‒ 32]. Thus, we believe that PLAT may inhibit thyroid cancer angiogenesis through VEGFa/VEGFR2.

## Discussion

The prognosis for PTC varies greatly because of its heterogeneity, from a 90% 10-year survival probability to a median of 3‒5 years [ 2, 4]. The heterogeneity of tumor is influenced by tumor cells and cells in the TME, suggesting that the clinical assessment remains challenging. As far as we know, the investigation of cells in the TME is far from adequate, which motivates us to investigate other cells in the TME. In this study, by combining the analysis of genome of a single cell from the GEO PTC datasets and bulk-RNA-seq from TCGA and WGCNA analysis, we specifically revealed that C10 belongs to EC and characterized 5 markers of EC associated with the progression of tumor. Furthermore, we built and verified a strong molecular signature consisting of 5 genes related to EC using GSE datasets for the first time, which can predict prognosis and treatment effectiveness in PTC patients. Using Cox regression, researchers are able to confirm that the presence of a signature associated with EC is an independent risk factor for OS. Subsequently, we constructed and validated a nomogram to enhance signature predictive ability and clinical applicability.

In this study, C10 cell cluster was identified to be significantly increased in tumor tissues. C10 belongs to EC, which actively affects tumor cells, promotes angiogenesis and plays a role in cancer progression [33]. More and more evidence shows that abnormalities of EC contribute to cancer progression by inducing cancer vascularization, intravasation of cancer cells, and CAF formation [34].

Although the research focusing on the influence of EC on the progression of TC is limited, EC has been identified to play an essential role in bladder tumor, prostate cancer, breast cancer and so on [ 35‒ 37]. In this study, we particularly found that EC in the TME had a strong relationship with the clinical stage of TC, which may provide a new perspective to the therapy of advanced TC. In addition, endothelial cells undergo a process called endothelial-mesenchymal transition (EndMT), which results in the loss of their intercellular connections. Numerous potential targets and medications offer fresh perspectives and ideas for clinical targeted treatment and tumor immunotherapy [38]. In tumors, EndMT is an important source of cancer-related fibrous cells (CAFs) which are known to promote tumor progression in many ways [39]. Endocrine cells play an important role in the initial stages of tumor migration. Studies have shown that tumor endocrin cells stimulate tumor cell migration by secreting a small repetitive protein polysaccharide rich in luminous acid called biglycan that interacts with tumor cells [40].

For the molecular pathways that the module makes TC more aggressive, there are 6 main up-regulated pathways: base excision repair, one carbon pool by folate, homologous, DNA replication, nucleotide excision repair, and mismatch repair. Base excision repair, homologous, nucleotide excision repair, and mismatch repair belongs to DNA repair pathways of DNA damage response (DDR) [ 41, 42], which safeguards the human genome against damage like DNA lesions, mutations, DNA strand breaks, interstrand, DNA protein links, and signals to cell cycle arrest, to allow time to repair, maintaining the stability of the genome and chromosomes [ 43, 44]. DDR dysregulation (upregulation or downregulation) can enhance the accumulation of DNA errors and genomic instability, two factors known to contribute to the development of cancer cells [ 42, 44], which not only causes tumor formation but also promotes tumor growth and progression. In this study, these pathways were found to be upregulated with the increase of risk score based on the 5 gene module. We propose that the module may increase the aggressiveness and risk of cancer through upregulating these pathways. DNA replication exists in most cancers, leading to genome instability and occurrence of cancerous cells [ 45, 46]. Thymidylate synthase (TSase) is essential to DNA replication because it is the sole enzyme in humans that catalyzes the continuous synthesis of nucleotides and is responsible for the *de novo* production of the DNA building component 2′-deoxy-thymidylate (dTMP) [47]. Thymidylate synthesis is regulated by folate mediated one-carbon metabolism (FOCM) [ 47‒ 49]. In this study, FOCM was found to be increased, which would provide the raw material for DNA replication and lead to the occurrence and progression of cancer. As for the 6 down-regulating pathways: the process of melanin synthesis and distribution is called melanogenesis [50]. A previous study showed that with the increase of melanogenesis, the subsequent accumulation of toxic melanin by-products would inhibit cell proliferation [51], which may be an explanation.

For the EC-related genes in this module, it has been established that the *CLDN5* gene is expressed in both endothelial and epithelial cells, and that the reduction of CLDN5 would decrease intercellular adhesion, leading to incomplete vascular endothelium and promotion of tumor metastasis [ 52‒ 54]. Yang *et al*. [55] proved that high expression of CLDN5 is associated with better relapse free survival (RFS) in breast cancer. A recent study showed that Hedgehog signaling-induced endothelial dysfunction is associated with the reduction of CLDN5 [56]. Hedgehog signaling regulates the vasculature through inducing the differentiation of hematopoietic and endothelial cells [57]. In our study, the risk score was found to be increased with the reduction of CLDN5 and the risk score is increased by downregulating hedgehog signaling pathway. We speculated that the reduction of CLDN5 may downregulate hedgehog signaling pathway, which causes endothelial barrier breakdown and increases the aggressiveness of tumor. Rudzika *et al*. [58] found that PLAT was differently expressed in human follicular thyroid carcinoma (FTC) tissues compared with normal tissues, suggesting that PLAT plays an active role in vascularization. Previous studies revealed that high expression of PLAT would lead tumor growth, such as lung cancer and ovarian cancer. However, according to our findings, PLAT overexpression could inhibit the migratory capacity of thyroid cancer cells. What’s more, PLAT in thyroid cancer cells can inhibit HUVEC proliferation and tube formation through VEGFa/VEGFR2, which is a new finding in thyroid cancer. Previous studies have found that VEGFR2 phosphorylation could further stimulate ERK signaling in breast cancer cells, in which cell proliferation, migration, invasion, and differentiation are triggered by the phosphorylation and activation of the mitogen-activated protein kinases 1 and 2 (MEK1/2), which in turn phosphorylates and activates the extracellular signal-regulated kinases 1 and 2 (ERK1/2) [ 59, 60]. Thus, we propose that PLAT may inhibit thyroid cancer cell proliferation and migration by suppressing the phosphorylation of VEGFR2 and further inhibiting the ERK signaling way ( Supplementary Figure S10). A recent study showed that TMEM47 is also screened as the prognostic signature for thyroid cancer and is beneficial to prognosis [61]. However, there is no study showing the biological role of this gene in the development and progression of thyroid cancer. Many tumor-cell characteristics, including stem-like self-renewal, EMT, radio- and chemo-resistance, and angiogenesis, have been linked to NOTCH4, and most research suggested that NOTCH4 is abnormally overexpressed during cancer development [62].

We further demonstrated that the module is associated with immune response. There were more Treg cells in the high-risk group than in the low-risk group, which suppresses effective tumor immunity and are often correlated with an undesirable prognosis in patients with cancer [63]. However, M2 macrophages were fewer in the high-risk group than in the low-risk group, which are dominant in human cancers and produce growth-promoting molecules that stimulate tumor growth effectively [64]. Although there was contradiction between the distributions of two immune cells in the group at high risk, which makes it hard to assess the efficiency of immunotherapy, high-risk PTC patients were found to have a much lower likelihood of responding to anti-PD-1 therapy using the TIDE algorithm. IMvigor210 cohort also showed that the ratio of CR/PR was lower in the group with high risk than in the group with low risk. Although these results suggested that the high-risk PTC patients have a lower chance of improving with immunotherapy, we can try to use immunotherapy as an adjuvant therapy. For example, anti-PD-1/PD-L1 therapy, as discovered by Gunda *et al*. [65], improves the efficacy of lenvatinib by modifying the immune microenvironment of murine anaplastic thyroid cancer. Case report by Cabanillas *et al*. [66] described a patient with advanced, locally recurrent ATC who was initially treated with trametinib and dabrafenib and then with the immunotherapeutic pembrolizumab (an anti-PD-1 antibody). A PR was reached, allowing surgical resection and chemoradiation. What’s more, Lu *et al*. [67] demonstrated that lenvatinib induced MDSC expansion and tumor infiltration only in ATC, indicating that the immunotherapy could play an important role in ATC. Interestingly, Pu *et al*. [21] proposed that PTC malignant thyrocytes could be divided into 3 types, among which the Pearson’s correlation between dedifferentiation-like cells and ATC cells was as high as 0.72 [21]. In another word, the prognosis of PTC with dedifferentiation-like cells was poor. Considering that our study analyzed the same PTC patients, we believe that the high-risk PTC patients may have dedifferentiation-like cells. At the same time, as mentioned above, combination of lenvatinib and immunotherapy can significantly improve the lenvatinib’s efficacy in ATC, we believe that combination of lenvatinib and immunotherapy may be beneficial for the high-risk PTC patients.

Nevertheless, there are several limitations in this study. Due to the fact that the tissue microarray verification cohort was a backwards study using publically accessible sequencing data, it had an insufficient sample size. This has to be validated in a big prospective clinical trial. More fundamental experimental studies, particularly of PLAT, are needed to elucidate the molecular pathways by which these linked genes affect patient prognosis and therapy responses. We only indicated that PLAT can inhibit thyroid cancer angiogenesis through VEGFa/VEGFR2 and other signaling ways also need to be explored.

In conclusion, we selected ECs as the crucial cell types using sc-RNA seq and constructed an EC-gene related module, which may assess the prognosis of PTC based on DNA repair pathways. The predictive accuracy of the model with numerous biomarkers would be higher than that of a single biomarker. Our findings also provide genetic evidence for future research paths on EC in PTC, suggesting that immunotherapy alone may not be effective for individuals at high risk of complications.

## Supporting information

23155Supplementary_data

## Supplementary Data

Supplementary Data is available at *Acta Biochimica et Biophysica Sinica* online.
