# Supplementary material for: Integration of scRNA and bulk RNA-sequence to construct the 5-gene molecular prognostic model based on the heterogeneity of thyroid carcinoma endothelial cell: Five-gene TC prognostic model
Source: Acta Biochim Biophys Sin (Shanghai). 2024 Jan 8;56(2):255–69. doi: 10.3724/abbs.2023254 (PMC10984871; doi:10.3724/abbs.2023254)
Supplement: 23155Supplementary_data [file 23155Supplementary_data.pdf]

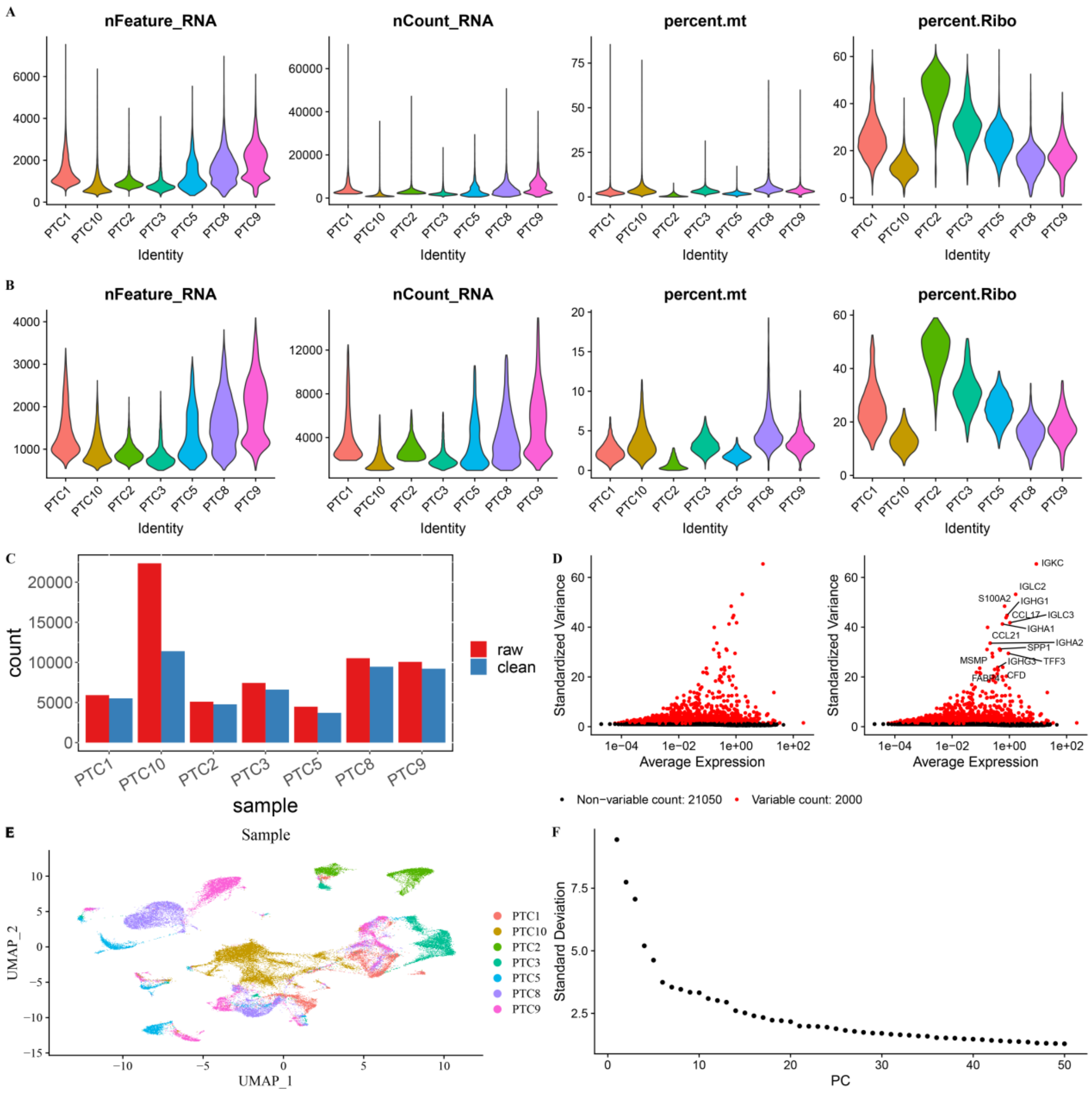

Supplementary Figure S1. Quality filtering (A) The relationship between the mRNA/UMI/mitochondrial content/rRNA content of each sample before filtering. (B) The relationship between mRNA/UMI/mitochondrial content/rRNA content of each sample after filtering. (C) Number of cells before and after filtration of the samples. (C) The average expression of genes before and after filtration of the samples. (E,F) The sample distribution map of PCA dimensionality reduction, colored by sample and the anchor point map of PCA.

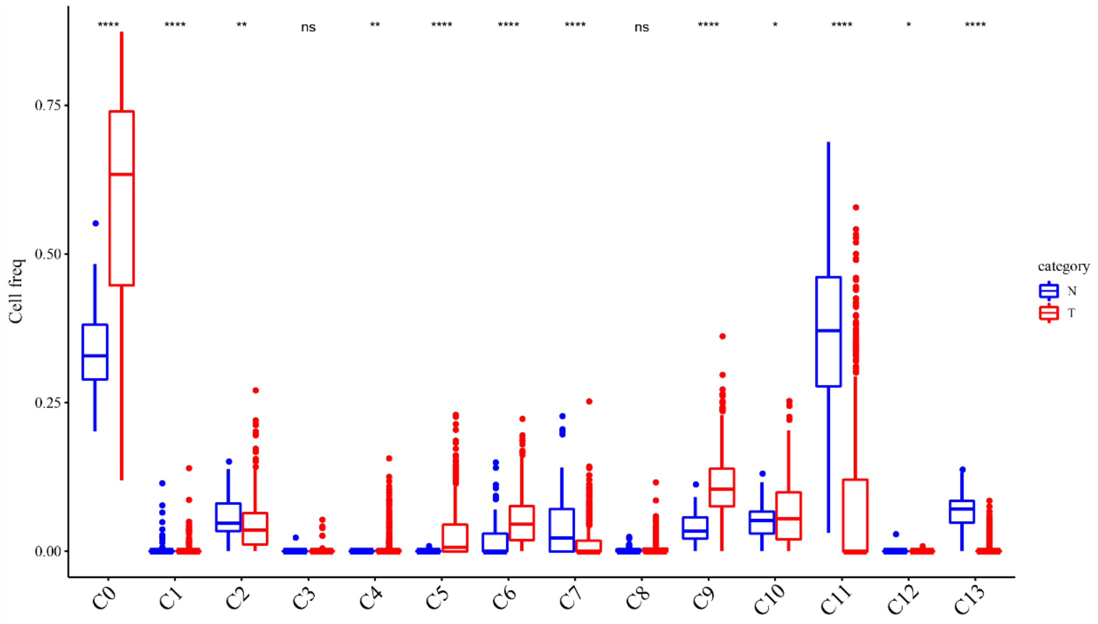

Supplementary Figure S2. Prediction and analysis of cell clusters in TCGA cohort Prediction of cell clusters of TCGA and comparison of the percentage of tumor cells and normal cells in each cell clusters. ****P<0.0001, ***P<0.001, **P<0.01, and *P<0.05.

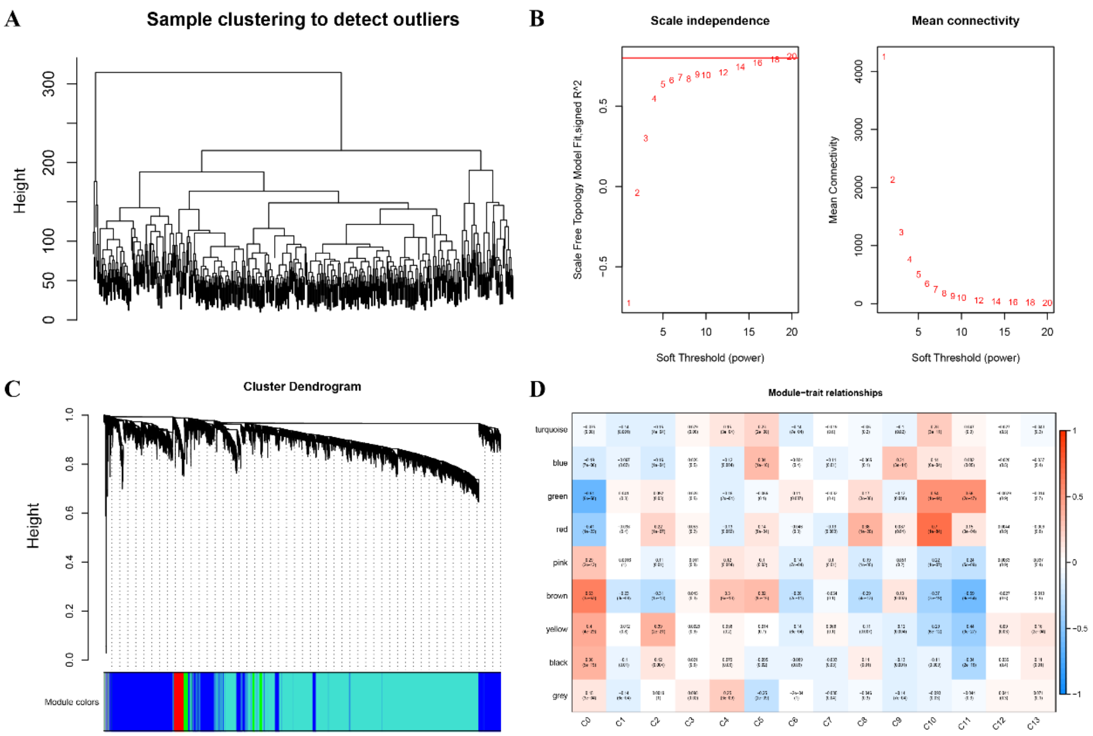

Supplementary Figure S3. WGCNA of genes in thyroid carcinoma (A) Clustering dendrogram of data from 487 thyroid cancer samples. (B) Analysis of the scale-free index for various soft-threshold powers and the mean connectivity for various soft-threshold powers. (C) Dendrogram of 11,386 genes. Nine coexpression modules were yielded. Each branch represents a gene, and each color represents a coexpression module. (D) Heatmap of the correlation between the module eigengenes and the marker genes of 14 cell clusters. Red represents positive correlation and blue represents negative correlation. The relationship of red module and C10 is the highest

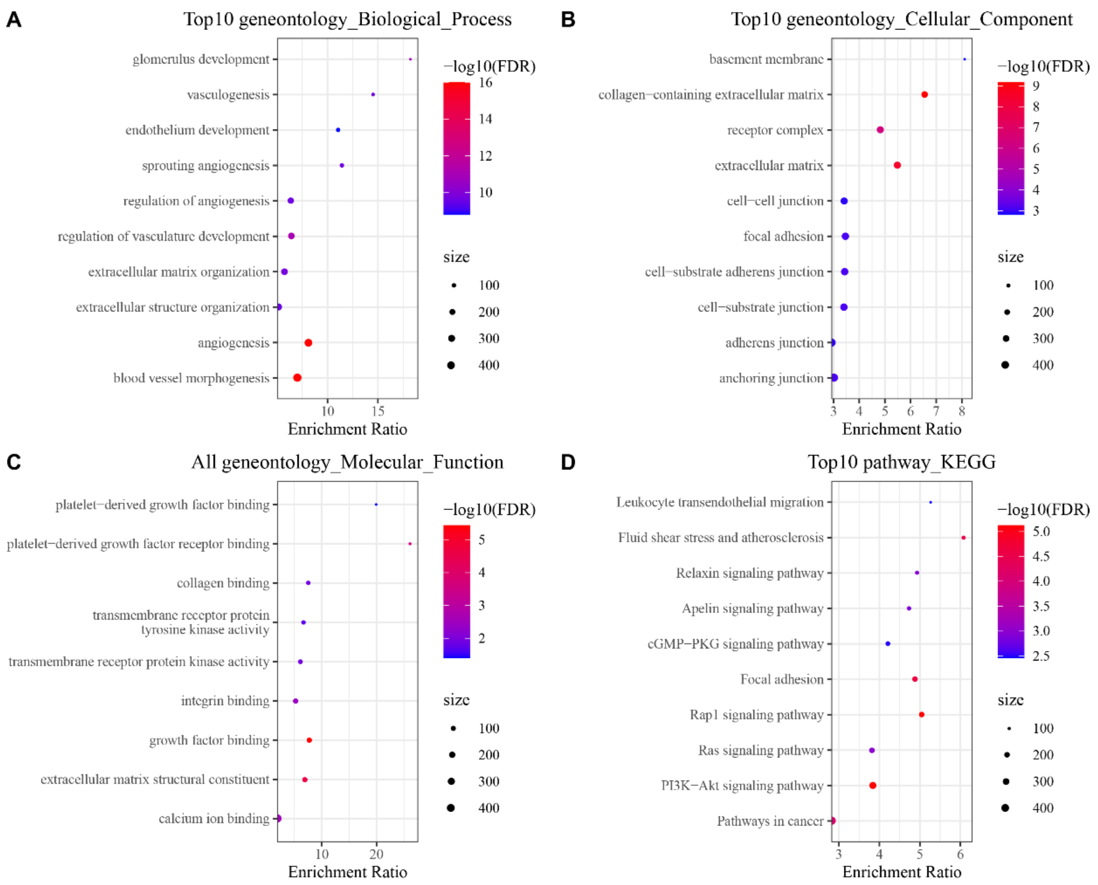

Supplementary Figure S4. Gene Ontology and KEGG enrichment analysis of the red module genes (A) Biological process analysis of top 10 gene ontology. (B) Cellular component analysis of top 10 gene ontology. (C) Molecular function analysis of top 10 gene ontology. (D) KEGG enrichment analysis of top 10 pathways. The term color represents the degree of enrichment. The term size represents the amount of enrichment. The x-axis shows the percentage of genes enriched.

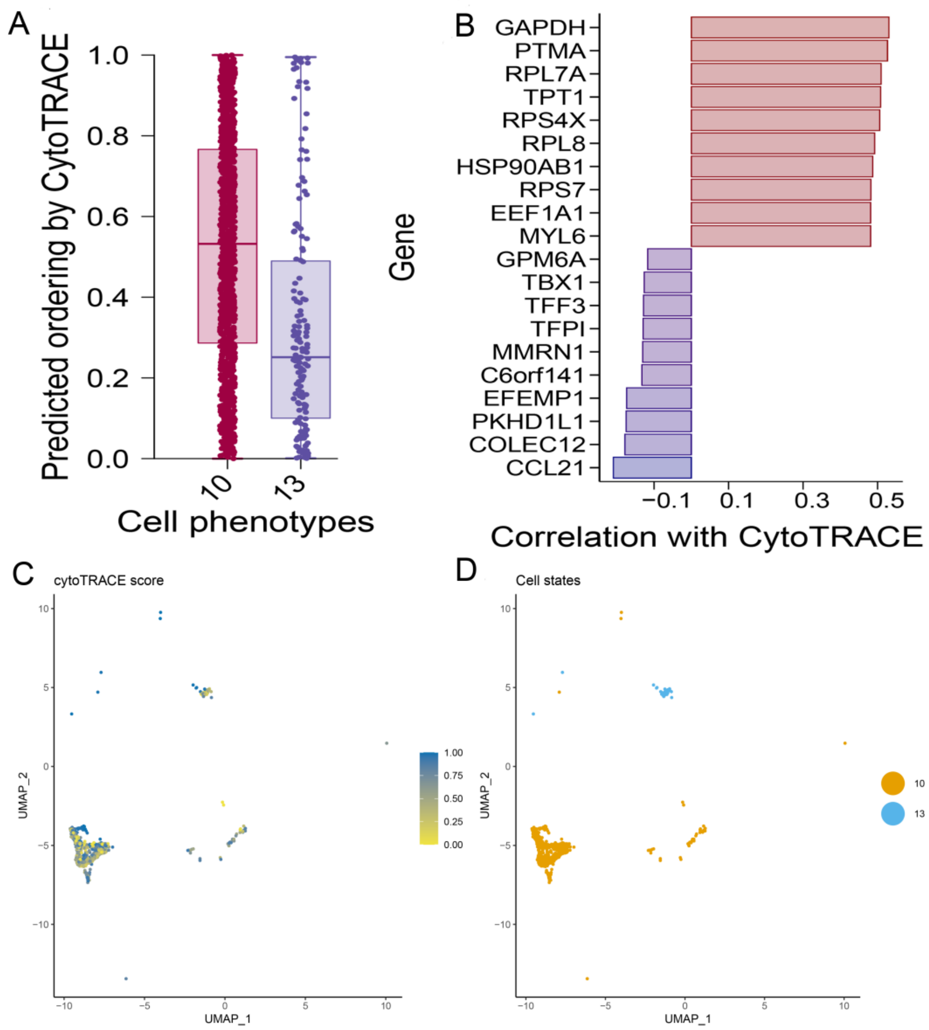

Supplementary Figure S5. Analysis of the developmental pseudotime trajectory of crucial cell clusters (A) Analysis of C10 and C13 belonging to Endothelial cells based on CytoTRACE. The cytoTRACE score of C10 is higher than C13. (B) Analysis the correlation of genes associated with differentiation and stem cell properties based on CytoTRACE. Red shows genes of low differentiation and blue shows genes of high differentiation. (C,D) Differentiation module on UMAP based on trajectory analysis using CytoTRACE. The degree of differentiation of C10 is low, whereas that of C13 is different.

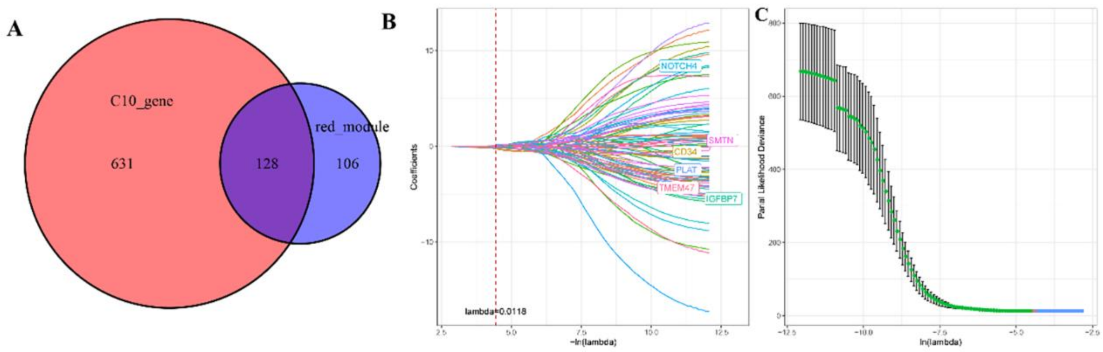

Supplementary Figure S6. Construction of the 5-gene molecular prognostic model (A) Venn diagram of the red module and marker genes of C10. (B) Determination of crucial genes by the LASSO analysis. (C) The optimal values of the parameter were determined by 10-round cross-validation.

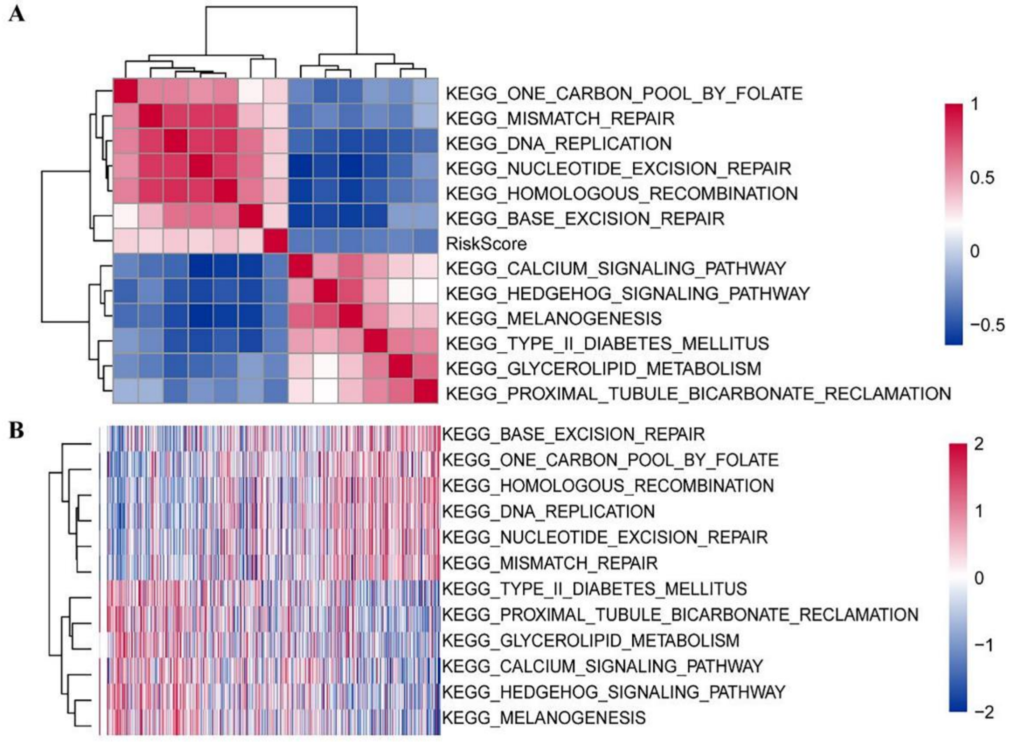

Supplementary Figure S7. Biological pathways potentially regulated by RS (A) Clustering KEGG pathways that the correlation with RS is greater than 0.3. (B) The relationship between crucial KEGG pathways and ssGSEA scores in all samples. The horizontal axis represents the samples, and the risk score increases successively from the left to the right.

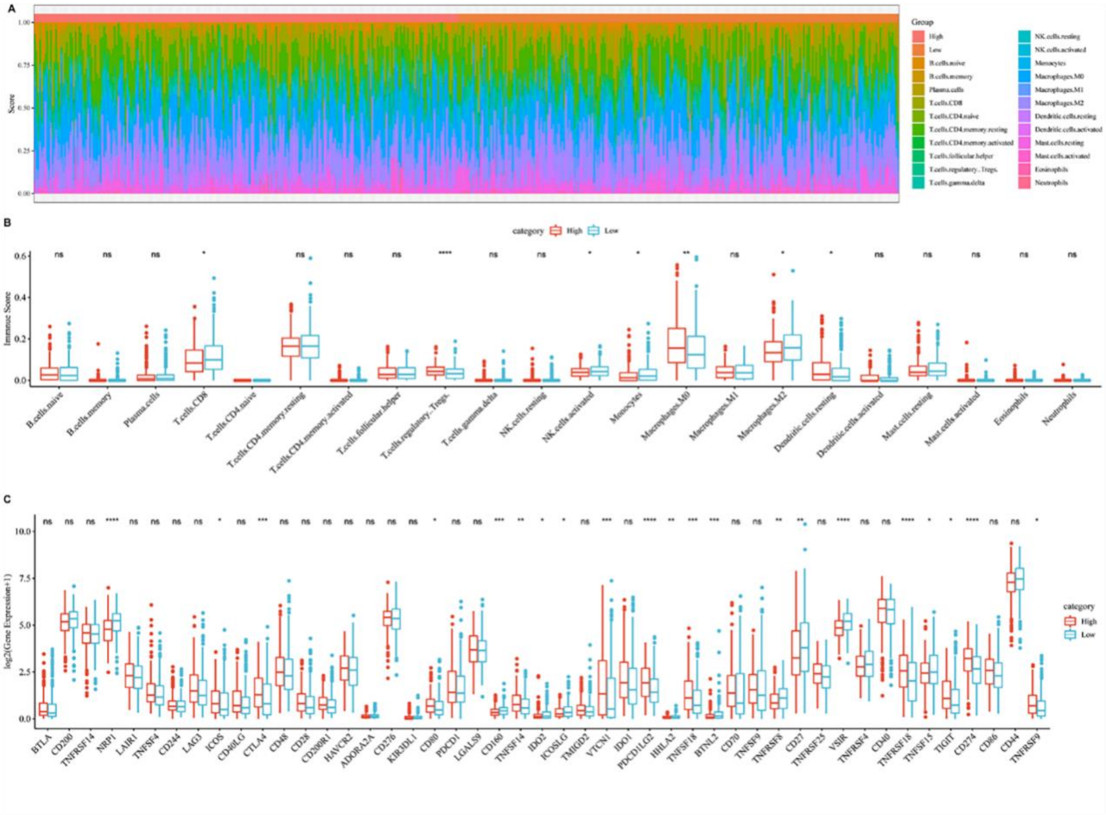

Supplementary Figure S8. Different distributions of immune cells in high-risk group and low-risk group (A) The ratio of 22 types of immune cells in the high-risk group and low-risk group. (B) The differences of in the high-risk group and low-risk group. (C) The differences of 47 immune checkpoints in the high-risk group and low-risk group.

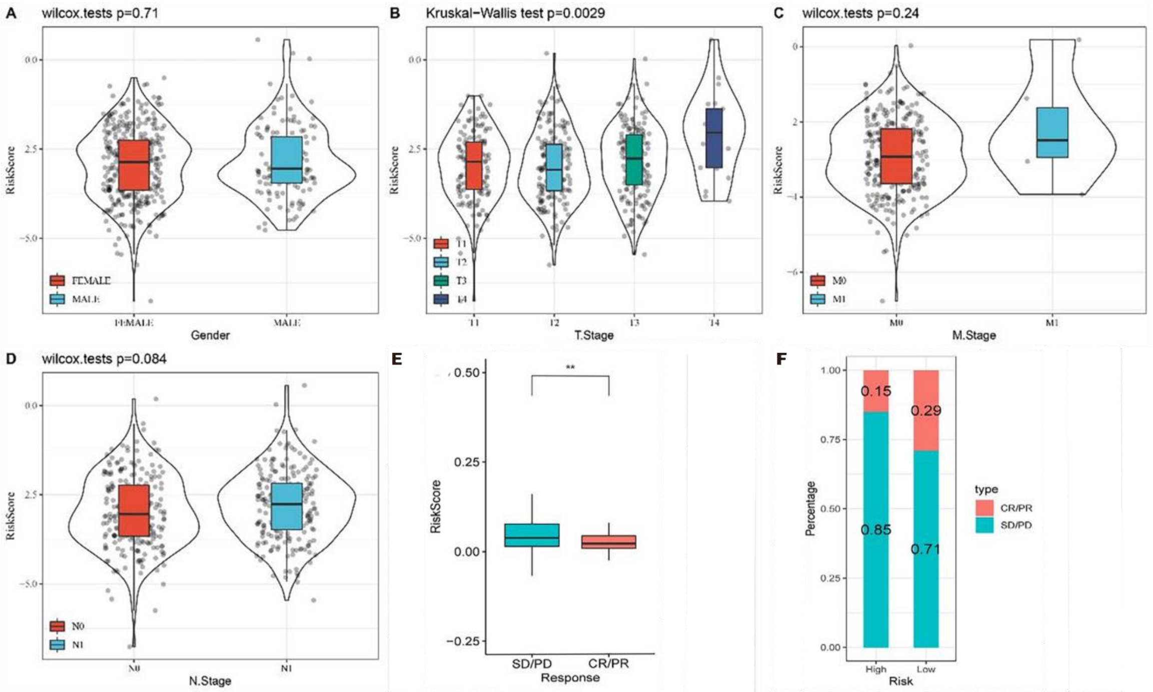

Supplementary Figure S9. The relationship between the 5-gene molecular prognostic model and immunotherapy (A) TIDE scores. (B) T-cell dysfunction scores. (C) T-cell rejection scores. (D) MDSC scores. (E) The risk scores in the CR/PR and SD/PD, ***P*<0.01. (F) The percentages of CR/PR and SD/PD in the high and low risk groups. CR: complete response; PR: partial response; SD: substance disease; PD: progressive disease.

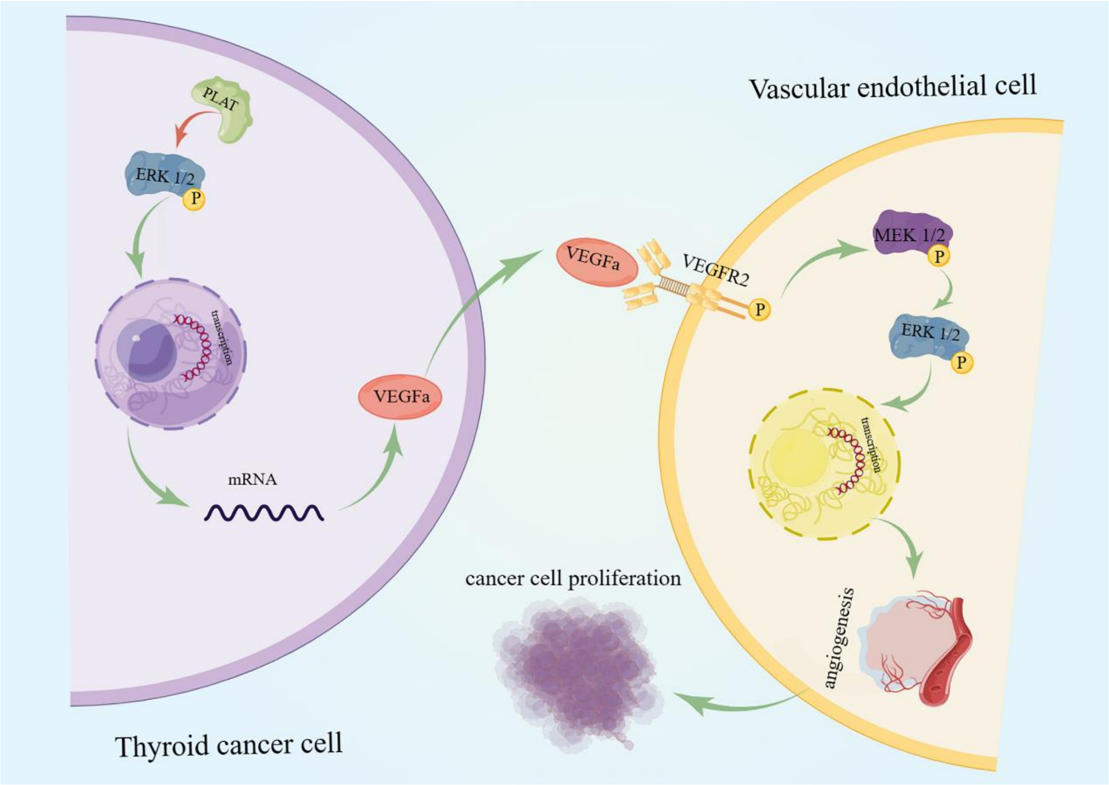

Supplementary Figure S10. Schematic diagram summarizing the signaling pathway through which PLAT inhibits thyroid cancer angiogenesis

Supplementary Table S1. Crucial 128 genes associated with survival

Name	*P* value	HR	Low 95%CI	High 95%CI
PLVAP	0.011641007	0.693205611	0.521470709	0.921497624
SPARCL1	0.001029099	0.668642909	0.525795625	0.850298707
FLT1	0.030011235	0.747927571	0.57534094	0.972285495
RAMP2	0.009825917	0.616906767	0.427534721	0.890159185
PLPP1	0.002737509	0.628767663	0.46414508	0.851778445
A2M	0.015586901	0.600836429	0.397608228	0.907939998
CALCRL	0.012940923	0.692333069	0.518070101	0.925212781
GNG11	0.021276656	0.670946595	0.477742127	0.942285195
AQP1	0.042694302	0.695241387	0.489179867	0.988104005
PECAM1	0.019849241	0.57648025	0.362651282	0.91638854
RAMP3	0.006462769	0.603765752	0.419914418	0.868112804
EGFL7	0.005520933	0.596332532	0.41391862	0.859136244
RNASE1	0.009089762	0.557368753	0.359262569	0.864715541
EMCN	0.000487217	0.588745259	0.437153855	0.792903861
EPAS1	0.00128343	0.583469342	0.420321683	0.809942688
CLDN5	0.000382843	0.529934676	0.373280209	0.752332308
EDNRB	0.035482328	0.778062747	0.615790183	0.983097254
CLEC14A	0.009333311	0.607490642	0.417195381	0.884585248

PODXL	0.026932916	0.690712222	0.497664052	0.958645439
KDR	0.025976858	0.749116259	0.58092372	0.966004916
ADGRL4	0.011362217	0.613178514	0.419875947	0.89547375
IGFBP7	0.000471967	0.513773016	0.353695322	0.746299697
RGCC	0.003410748	0.64823809	0.484970281	0.866470871
SLCO2A1	0.002596345	0.643342013	0.482821497	0.85722974
TCF4	0.020895665	0.58605838	0.372421109	0.922247468
PTPRB	0.003366097	0.566417546	0.387368532	0.828226377
CD34	0.032281228	0.682368886	0.480913426	0.968214385
TMEM88	0.004135117	0.664407122	0.502427522	0.878607969
COL15A1	0.033027858	0.710739869	0.519243208	0.972860414
VWA1	0.012704373	0.61626491	0.421129577	0.901818489
TM4SF18	0.01221424	0.657223023	0.473301969	0.91261421
APOLD1	0.011372012	0.735602544	0.579930833	0.933061448
PDGFD	0.027788618	0.716061677	0.531792197	0.964181739
CD93	0.028451702	0.667735062	0.465268387	0.958307345
TMEM204	0.003209874	0.608394122	0.437167003	0.846686517
ABCG2	0.000252874	0.535460502	0.383205689	0.74820901
MMRN2	0.009414801	0.606025768	0.415252214	0.884443765
LDB2	0.003562525	0.612345414	0.440305988	0.851605285

CYYR1	0.001827709	0.56170305	0.390833199	0.80727614
GRB10	0.008317645	0.571575866	0.377265873	0.865964813
THBD	0.010332305	0.64378954	0.459804696	0.901393518
FAM167B	0.014284894	0.596913637	0.395043879	0.901940035
PALMD	0.019552423	0.566779322	0.351899079	0.912871953
NOTCH4	0.031303281	0.679368639	0.477835232	0.965901459
PLAT	0.000150302	0.57096192	0.427324381	0.762880679
JAM2	0.000254468	0.463430944	0.306906043	0.699784982
FZD4	0.001988753	0.535651339	0.360595448	0.795690458
ROBO4	0.016608516	0.651483763	0.4588038	0.925081903
NOSTRIN	0.008277145	0.577167329	0.383819086	0.867914435
APLNR	0.039965046	0.724201932	0.532282771	0.985319208
TEK	0.0054893	0.621887523	0.444741409	0.86959317
ACVRL1	0.00930802	0.560168806	0.361936326	0.866973191
PIK3R3	0.003443247	0.538969066	0.356204401	0.815508325
LIMS2	0.003061886	0.511764851	0.328493019	0.797287151
MYCT1	0.003612117	0.617019105	0.445726185	0.854140028
CDA	0.045699622	0.673648789	0.457242325	0.992477437
LMO2	0.000943625	0.470047334	0.30048208	0.735300075
ZEB1	0.048727887	0.671975068	0.452553522	0.997783622

TMEM150C	0.004549895	0.567624749	0.38385722	0.839369013
MCF2L	0.012998479	0.53199433	0.32331173	0.875371788
CLEC3B	0.003138159	0.675637998	0.520865093	0.87640103
ACE	0.015348039	0.63111535	0.434998258	0.915650988
SHANK3	0.008595935	0.592676565	0.401205628	0.875524882
TMEM47	0.000412349	0.582909162	0.432046918	0.786449521
RASIP1	0.005078804	0.568444595	0.382908589	0.843880932
ADCY4	0.022087864	0.572124222	0.354672346	0.922897231
ERG	0.010000188	0.575650949	0.378148584	0.876306375
SMTN	0.049292799	0.660348701	0.436620966	0.998716144
FGD5	0.010783512	0.556510447	0.354660542	0.873240298
ARHGEF15	0.006231222	0.563687172	0.373809034	0.850014844
SOX17	0.030743295	0.596954088	0.373824301	0.953266498
NRN1	0.000677158	0.445753321	0.279725558	0.710324879
NES	0.014249285	0.676137752	0.49444353	0.924599539
AFAP1L1	0.02663669	0.6438852	0.436282918	0.950273627
SPRY4	0.043193022	0.688712875	0.479769846	0.988652012
GIMAP7	0.001166237	0.581192672	0.418850302	0.806457391
GIMAP8	0.000791827	0.552369165	0.390544456	0.781247025
PDGFB	0.029153026	0.629433102	0.415249288	0.954092016

SH2D3C	0.007737108	0.591920336	0.402411374	0.870675402
SOCS2	0.000434958	0.454234232	0.292642398	0.70505415
GIMAP6	0.003078252	0.584520896	0.409616052	0.834109592
IFITM2	0.040716508	0.656294318	0.438453103	0.98236785
KLF2	0.00067224	0.601741793	0.449028137	0.806393086
GIMAP1	0.000289901	0.412585365	0.255612342	0.66595643
GRASP	0.014389712	0.610243664	0.410890116	0.906318539
PLAC9	0.010311612	0.672049523	0.496050305	0.910493467
LRRC8C	0.006531647	0.462195573	0.265028198	0.806045355
FLI1	0.012670653	0.529822434	0.321540765	0.873020911
